# Efficacy of omalizumab in adult patients with allergic bronchopulmonary aspergillosis: a multicentre study in China

**DOI:** 10.1007/s10238-023-01267-y

**Published:** 2024-01-19

**Authors:** Peixv Chen, Yali Yu, Li He, Chunyi Zhang, Yiting Li, Di Wu, Ying Chen, Ran Wang, Guopeng Xu, Chao Cao

**Affiliations:** 1 Department of Respiratory and Critical Care Medicine, Key Laboratory of Respiratory Disease of Ningbo, The First Affiliated Hospital of Ningbo University, 59 Liuting Road, Ningbo, 315010 China; 2 School of Medicine, Ningbo University, Ningbo, China; 3 Department of Respiratory and Critical Care Medicine, Ningbo Hospital of Traditional Chinese Medicine, Zhejiang, China; 4 Department of Respiratory and Critical Care Medicine, Jingzhou Hospital Affiliated to Yangtze University, Hubei, China; 5 Department of Respiratory and Critical Care Medicine, Shaoxing People’s Hospital, Zhejiang, China; 6 Department of Respiratory and Critical Care Medicine, The Affiliated Suzhou Hospital of Nanjing Medical University, Jiangsu, China; 7 Department of Respiratory and Critical Care Medicine, The First Affiliated Hospital of Anhui Medical University, Anhui, China

**Keywords:** Allergic bronchopulmonary aspergillosis, Asthma, Monoclonal anti-IgE, Retrospective study

## Abstract

Despite conventional glucocorticoid and antifungal therapy, acute exacerbation and hospitalization occur frequently in patients with allergic bronchopulmonary aspergillosis (ABPA). Whether omalizumab is an effective and safe treatment for adult patients with ABPA complicating asthma. Patients with ABPA complicating asthma who were treated with omalizumab from October 2019 to May 2023 were collected from five tertiary hospitals and evaluated. The frequencies of acute exacerbation and hospitalization; the number of eosinophils; the total IgE levels; and the average monthly medical dosages after 3, 6, and 12 months of omalizumab treatment were analysed, and the data before and after treatment (up to one year) were compared. The efficacy and safety of omalizumab treatment were assessed. In total, 26 patients were enrolled. The average monthly glucocorticoid dosage significantly decreased (median 0 vs*.* 24 mg/m) after 6 months of omalizumab treatment compared with 3 months; 73.68% of patients discontinued glucocorticoids after ≤ 12 months of treatment. Similarly, the average monthly dosage of antifungal agents was significantly decreased (median 0 vs*.* 3.49 g/m) after 12 months of treatment compared with 3 months. The average monthly glucocorticoid dosage (median 213.75 vs*.* 65.42 mg/m, *P* = 0.002) and the frequency of acute exacerbation (median 0.94 vs*.* 0.44 events, *P* = 0.033) were considerably reduced after omalizumab treatment. Omalizumab is effective in reducing the frequency of acute exacerbation and the necessary dosage of glucocorticoids in adult patients with ABPA complicating asthma. Patient age and BMI may affect the efficacy of treatment.

## Introduction

Allergic bronchopulmonary aspergillosis (ABPA) is a complex immunoreaction mediated by *Aspergillus* antigens and affects approximately 1–5% of patients with asthma [1]. The conventional way of treating ABPA is with cortisol hormones and antifungal agents, with the former often being used to suppress the immunoreaction [2, 3]. Such treatment aims to slow disease progression and control inflammation. However, it has also resulted in many patients becoming hormone dependent and suffering adverse effects from prolonged hormone use. Antifungal agents may provide an alternative to hormones [4]. By way of justification, previous studies have illustrated that the combined use of glucocorticoids and antifungal triazoles reduces the frequency of acute exacerbation more than the former alone [5]. Additionally, it is worth noting that the frequency of acute exacerbation and hospitalization is still not low, suggesting the need for additional treatment [6].

Elevated IgE levels are one of the acknowledged diagnostic criteria and markers of ABPA disease activity [7]. Omalizumab, a humanized IgG1 antibody, binds to free IgE in the blood to form a biologically inert complex [8]. Studies have demonstrated that omalizumab can reduce the number of instances of exacerbation and relieve symptoms when used in the treatment of asthma [9]. Nevertheless, for patients with ABPA complicating asthma, only a few case reports and series have described the use of omalizumab and have shown the possible mechanism of omalizumab treatment [10]. Moreover, most studies have not compared the exact frequency of acute exacerbation before and after treatment, and few have examined the dosage changes in asthma medications other than antifungal agents and hormones or performed correlation analyses [11–13].

This retrospective study was aimed at assessing the efficacy and safety of omalizumab in adult patients with ABPA complicating asthma. The frequencies of acute exacerbation and hospitalization and the dosages of glucocorticoids and other asthma medications were investigated in patients treated with omalizumab.

## Methods

### Study design and participants

This study enrolled adult patients with ABPA complicating asthma who were treated with omalizumab between October 2019 and May 2023 in multiple tertiary hospitals. The patients all met the following criteria [3, 13]:

- They were all diagnosed with asthma;
- None of them had been diagnosed with cystic fibrosis (CF);
- Patients had serum IgE antibodies against *Aspergillus* or immediately reacted to aspergilli in skin tests;
- Total serum IgE levels were increased (IgE > 417 IU/mL);

Additionally, we ascertained that each participant met at least six of the Rosenberg–Patterson criteria.

Furthermore, the enrolled patients had been suffering from asthma for at least one year. Each patient’s dosage of omalizumab was determined by the individual’s IgE level and weight. (The maximum dosage was 600 mg/ every two weeks).

### Outcome

The demographic and clinical characteristics were recorded in patient files. After the patients started omalizumab treatment, the eosinophil counts; the total IgE levels; the frequencies of acute exacerbation and hospitalization; and the dosages of clinical medications (including glucocorticoids, antibiotics, antifungals, and *β* receptor agonists) at 3, 6, and 12 months were all recorded. The proportion of patients who stopped glucocorticoids at each stage was also tracked.

Acute exacerbation was defined as worsening respiratory tract symptoms (dyspnoea, wheezing, severe cough, or increased expectoration) that required the temporary use of additional hormones and agents for control, followed by a return to the normal dosage. In addition, the efficacy of omalizumab treatment was assessed by comparing the data before and after a course of treatment lasting no longer than one year. If the time was shorter than one year, the data were compared before and after for an equal period; if drug withdrawal or adverse reactions occurred during the treatment, they were also recorded for assessment.

### Statistical analysis

Statistical analyses were performed with *SPSS 27.0, R 4.2.2,* and *GraphPad Prism 8*. Data with normal distributions were expressed as the minimum (min)–maximum (max), while those with non-normal distributions were expressed as the median (quartiles). A matched-samples *t* test was used to compare two paired groups with normal distributions; if the data were not normally distributed, Wilcoxon’s signed-rank test was used. The Kruskal‒Wallis H test was used to assess the differences between independent groups.

The correlations between the average monthly dosages of the above four drugs, the frequencies of hospitalization and acute exacerbation, age, BMI, and the duration of asthma in omalizumab treatment were analysed with the Spearman or Pearson correlation coefficient. The former was used for non-normal data; otherwise, the latter was used. When the absolute correlation coefficient was between 0.2 and 0.4, the groups were regarded as having a weak correlation; coefficients between 0.4 and 0.7 were considered to indicate a moderate correlation; and coefficients above 0.7 were considered to indicate a strong correlation. If the frequency of missing values was < 10%, the average value of the variable was substituted for the missing values. The missing values of variables with loss rates between 10 and 50% were interpolated by multiple imputation. Variables with > 50% missing values were excluded.

All values of *P* were calculated from two-tailed tests, and a *P* value less than 0.05 was considered significant.

## Results

### Baseline characteristics

**Fig. 1:**
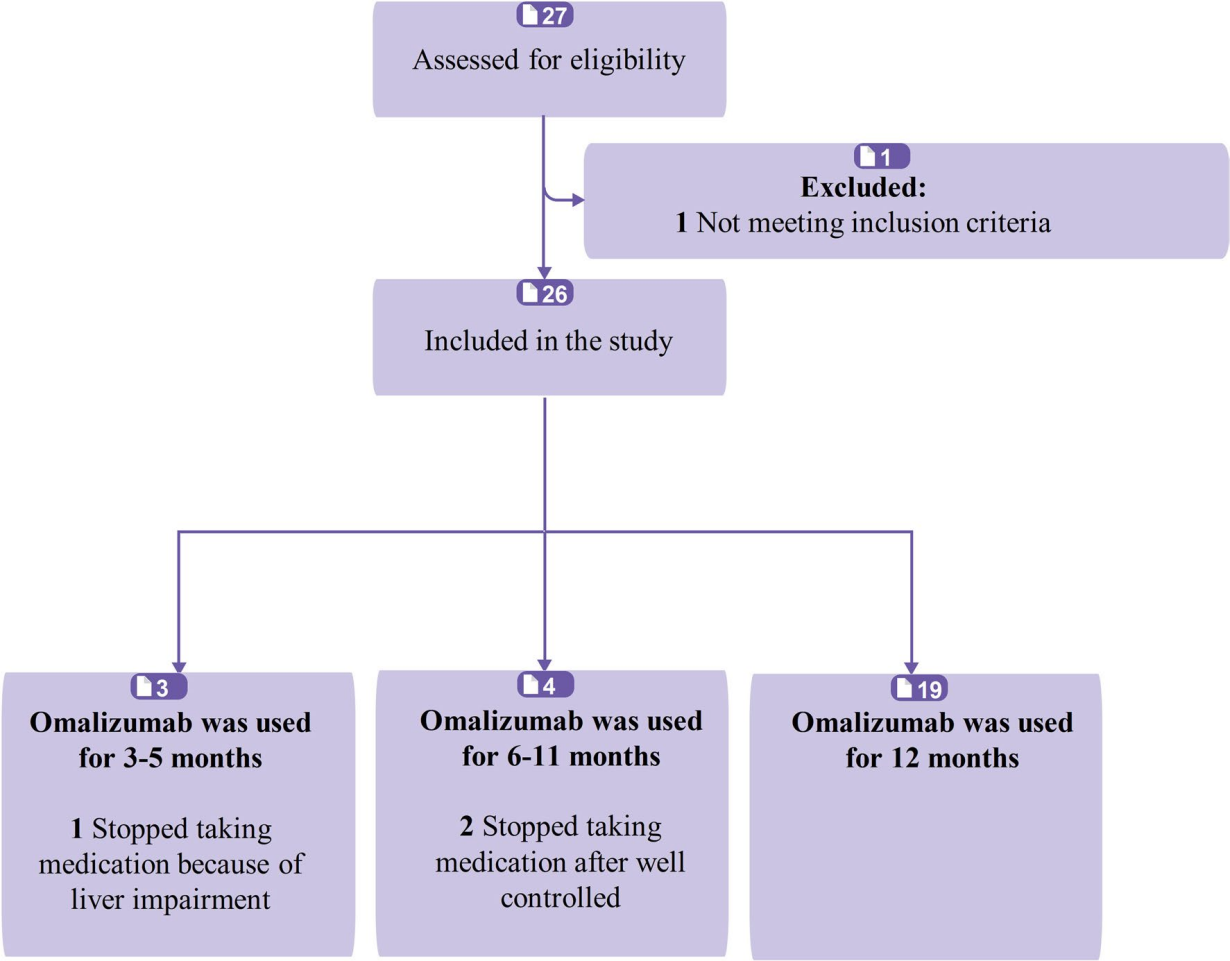
Participants’ screening flowchart

The patient screening process and treatment time are shown in Fig. 1. The demographic and clinical characteristics of the enrolled patients before omalizumab treatment are shown in Table 1. Among 26 adult patients with ABPA complicating asthma, 16 (61.5%) were male, and 10 (38.5%) were female. The average age was 52.35 ± 14.89 years (mean ± SD), (min–max, 20–75); the average BMI was 23.87 ± 3.20 (mean ± SD), (min–max, 20.02–33.22); and 22 (84.6%) of the participants had never smoked. Their average duration of asthma was 22.58 ± 15.21 years (mean ± SD), (min–max, 1–52); 11 (42.3%) of them were diagnosed with bronchiectasis after HRCT; and their fortnightly dosage of omalizumab was 467.31 ± 171.43 mg (mean ± SD), (min–max, 150–600).

**Table 1:** Baseline characteristics of enrolled participants

Index	
Males, n	16 (61.5%)
Age, y	52.35 ± 14.89 (20, 75)
BMI	23.87 ± 3.20 (20.08, 33.22)
None of a smoking history	22 (84.6%)
Total IgE, IU/mL	1916.58 ± 2083.15 (418, 10,800)
Blood eosinophils. *10^9^/ L	0.24 ± 0.24 (0.06, 0.76)
Dosage of omalizumab (once every two weeks), mg	467.31 ± 171.43 (150, 600)
Bronchiectasis in HRCT	11 (42.3%)
Duration of asthma, y	22.58 ± 15.21 (1, 52)

*HRCT* high resolution CT

### Changes in indicators with duration of omalizumab treatment

**Fig. 2:**
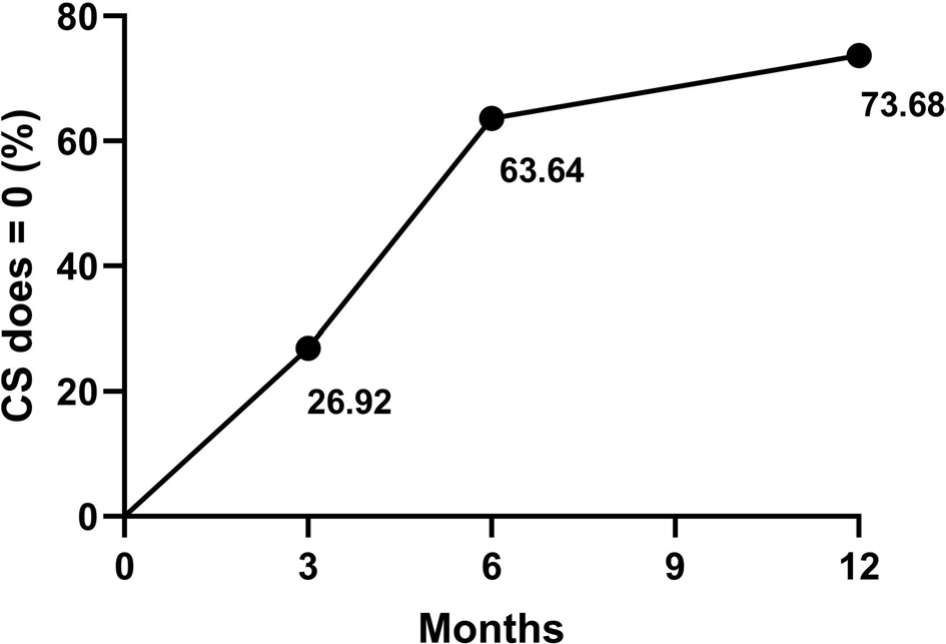
Changes of glucocorticoid usage over time after omalizumab treatment

The clinical characteristics, the frequencies of acute exacerbation and hospitalization, and the dosages of various drugs in patients treated with omalizumab at 3, 6, and 12 months were recorded (Table 2). The eosinophilic counts, total IgE, and mean monthly use of long-acting *β* agonists (LABAs) increased over the course of treatment, while acute exacerbations; hospitalizations; and monthly mean use of glucocorticoids, antibiotics, and antifungal agents decreased. Furthermore, the proportion of patients who had discontinued glucocorticoids rose over time (Fig. 2).

**Table 2:** Data of participants treated with omalizumab in 12-month follow-up

Time	3 M (26)	6 M (23)	12 M (19)
Eosinophil count, *10^9^cells/µL	0.23 ± 0.21 (0.01,0.55)	0.26 ± 0.23 (0.01,0.95)	0.39 ± 0.27 (0.08,1.3)
Total IgE, IU/mL	1488.31 ± 1169.47 (171,5004)	1750.10 ± 1805.87 (121,6289.2)	2302.34 ± 1885.09 (328,7787.7)
Asthma exacerbation, n	0.27 ± 0.53 (0,2)	0.17 ± 0.39 (0,1)	0.05 ± 0.23 (0,1)
Hospitalizations for ABPA*, n	0.46 ± 0.65 (0,2)	0.52 ± 0.79 (0,3)	0.16 ± 0.37 (0,1)
Dose of GC, mg/m	24 (0.08,390)	0 (0,37.14)	0 (0,0)
Dose of antifungal agents, g/m	3.49 ± 6.33 (0,23.8)	1.23 ± 2.99 (0,12.2)	0.00 ± 0.02 (0,0.07)
Dose of antibiotics, g/m	3.06 ± 8.73 (0,40.5)	3.64 ± 8.51 (0,28.5)	0.83 ± 1.82 (0,7.67)
Dose of LABA, mg/m	0.09 (0,0.27)	0.09 (0,1.23)	0.27 (0.05,3.49)

*M* months, *GC* glucocorticoids, *LABA* Long-acting *β* agonists, *ABPA* allergic bronchopulmonary aspergillosis

**Fig. 3:**
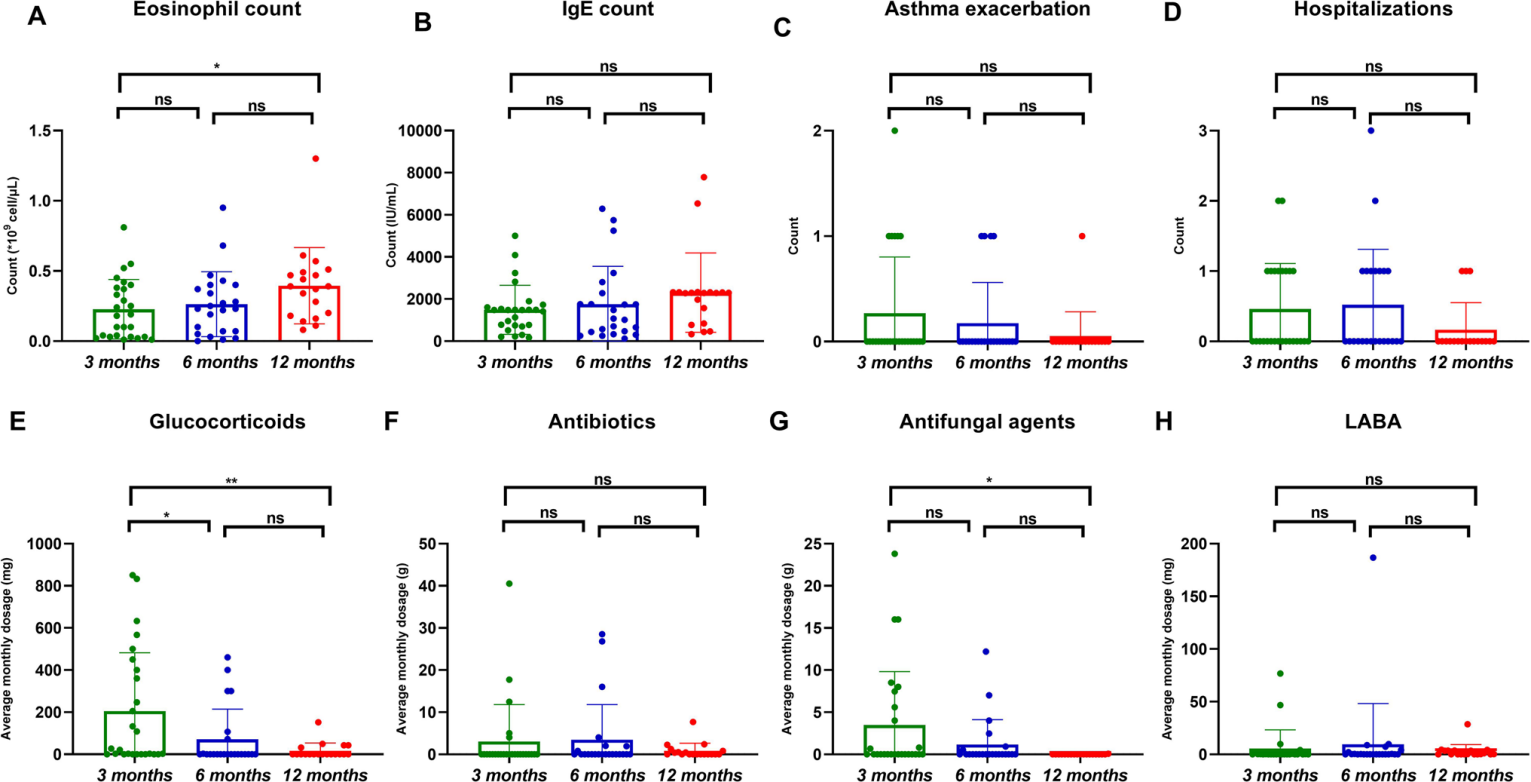
The changes of each index after 3, 6, and 12 months of treatment with omalizumab. **A** Eosinophil count, **B** IgE count, **C** Asthma exacerbations, **D** Hospitalization, **E** Glucocorticoids, **F** Antibiotics, **G** Antifungal agents, **H **LABA (Long-acting *β* agonists). Statistical significance was tested by Kruskal–Wallis test. *P* < 0.05 was considered statistically significant, *P < 0.05, ** *P* < 0.01, ***P < 0.001

The average monthly glucocorticoid dosage significantly decreased at 6 months (IQR: 0, 0–37.14 mg/m) and 12 months (IQR: 0, 0–16 mg/m) compared with 3 months (IQR: 24, 0.08–390 mg/m). However, there was no significant change between 6 and 12 months. The average monthly dosage of antifungal agents decreased distinctly after 12 months of omalizumab treatment compared with 3 months, while the eosinophil count was on the contrary. (Fig. 3).

**Fig. 4:**
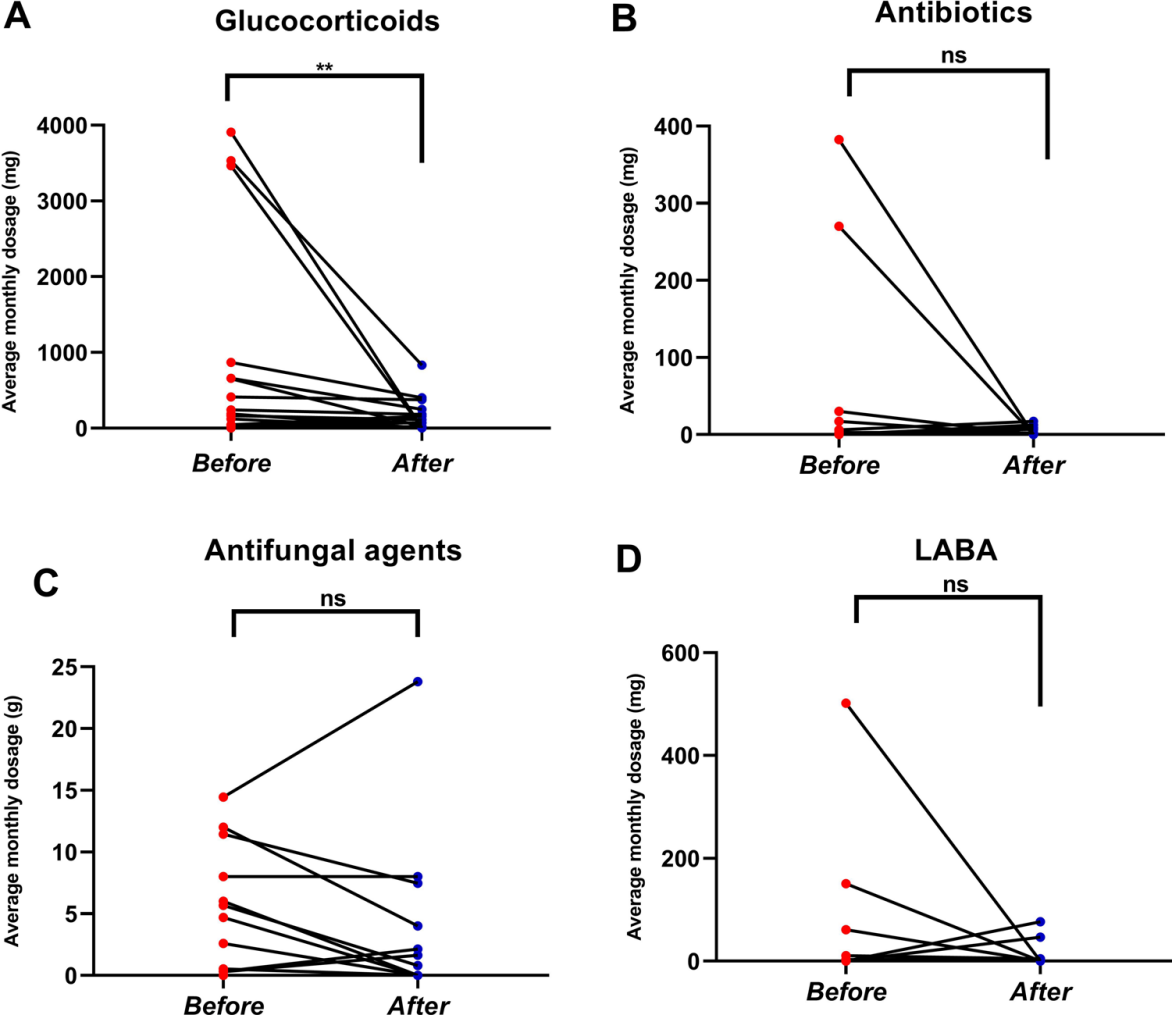
The changes of each index before and after omalizumab treatment for the same time. **A** Glucocorticoids, **B** Antibiotics, **C** Antifungal agents, **D** LABA (Long-acting *β* agonists). Statistical significance was tested by Wilcoxon’s signed-rank test. *P* < 0.05 was considered statistically significant, *P < 0.05, ** *P* < 0.01, ***P

After treatment with omalizumab, the average monthly dosage of glucocorticoids was lower than before by a remarkable margin (median 213.75 vs*.* 65.42 mg/m, *P* = 0.004), and similar results were observed in the frequency of acute exacerbation (median 0.94 vs*.* 0.44 events, *P* = 0.033). However, no significant difference in the average monthly dosage of antibiotics, antifungals, or LABAs or the frequency of hospitalization were found after treatment with omalizumab (Table 3 and Fig. 4).

**Table 3:** Data comparison before and after omalizumab treatment

	Pre-treatment	Aft-treatment	*P*
Dose of GC, mg/m	213.75 (63.75, 815.87)	65.42 (4.07, 230)	0.002
Dose of antibiotics, g/m	1.04 (0, 14.33)	0 (0, 5.60)	0.397
Dose of antifungal agents, g/m	1.56 (0, 7.5)	0 (0, 3.53)	0.203
Dose of LABA, mg/m	0.2 (0, 8.88)	0.16 (0, 3.45)	0.861
Asthma exacerbations, no	0.94 ± 0.85 (0, 3)	0.44 ± 0.73 (0, 2)	0.033
Hospitalization for ABPA, no	0.81 ± 0.83 (0, 3)	0.69 ± 0.95 (0, 3)	0.405

*GC* glucocorticoids, *LABA* long-acting *β* agonists, *ABPA* allergic bronchopulmonary aspergillosis

### Correlation analysis

**Fig. 5:**
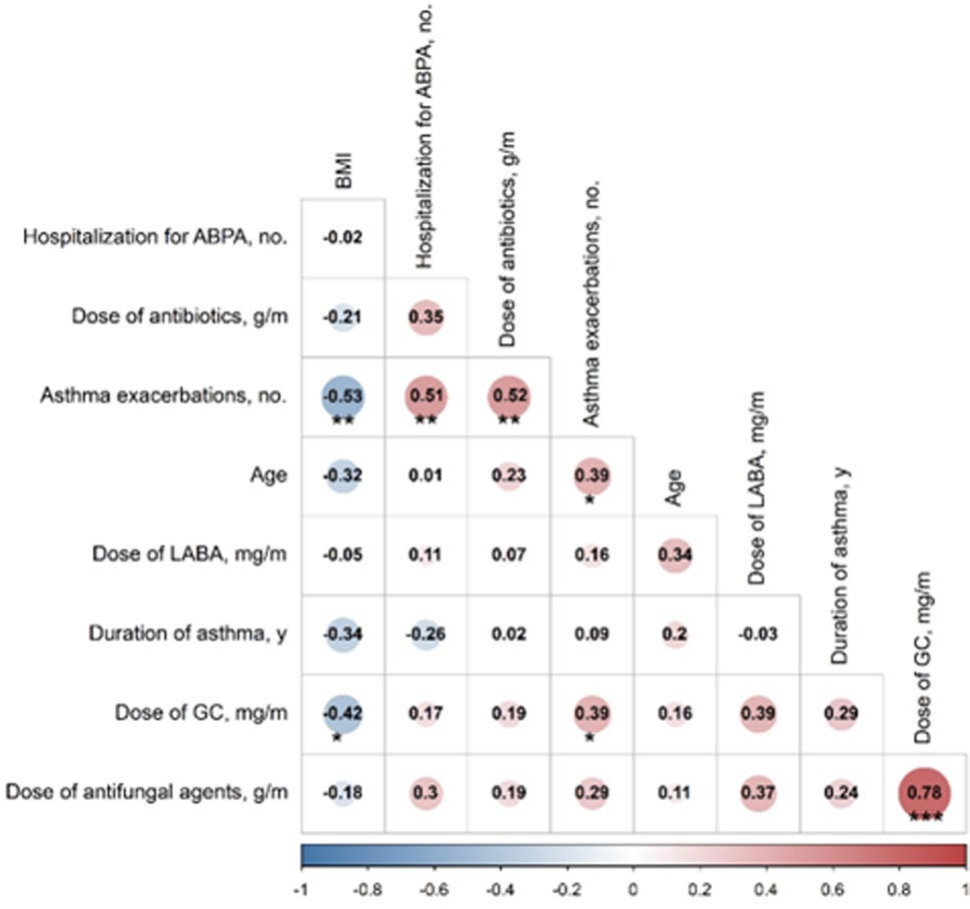
Correlation analysis. *GC* glucocorticoids, *LABA* long-acting *β* agonists, *ABPA* allergic bronchopulmonary aspergillosis. *Note: P* < 0.05 was considered statistically significant, *P < 0.05, ** *P* < 0.01, ***P < 0.001

The average monthly dosage of glucocorticoids had a moderate negative correlation with BMI (*r* = -0.42, *P* = 0.03). Moreover, the frequency of acute exacerbation showed a moderate positive correlation with age (*r* = 0.39, *P* = 0.047), and it had a moderate negative correlation with BMI (*r* = -0.53, *P* = 0.006). There was no significant correlation between age, BMI, and the duration of asthma (Fig. 5).

### Missing value processing

The data after interpolation were used for the above analysis, and the missing variables are shown in Appendix Table 4. There was no significant difference between the data before and after interpolation (Appendix Table 5).

### Safety

The safety of omalizumab treatment should be noted. In this study, one patient discontinued the treatment due to liver function impairment, but the other patients experienced no severe adverse effects. Two patients discontinued omalizumab and glucocorticoids after 6 months when they felt their symptoms had improved.

## Discussion

To our knowledge, this was the largest sample size study to date to assess the impact of omalizumab in patients with ABPA complicating asthma. The findings from our study showed that omalizumab treatment in adult patients with ABPA complicating asthma was effective in reducing the use of LABA, antifungal agents, antibiotics, and particularly glucocorticoids. Specifically, over 60% of patients discontinued glucocorticoids only after 6 months, and its average monthly dosage decreased markedly—more than 50% overall—after treatment (the median decreased from 213.75 to 65.42 mg/m). Moreover, the frequency of acute exacerbation was significantly reduced and clinical symptoms relieved in patients with ABPA complicating asthma who were treated with omalizumab. This study also suggested that adult patients who were in the younger age group (between 20 and 75 years) and who had a higher BMI (20.08 to 33.22) might receive greater benefits from the treatment.

It has been acknowledged that ageing causes progressive decline in almost all body systems and functions. However, a study involving numerous participants noted that the total IgE level increased with age, peaking in the subgroup over 85 years old [14]. It has also been noted that asthma is strongly correlated with the total serum IgE level, with a high IgE level leading to sensitization and exacerbating asthmatic symptoms owing to increased airway responsiveness, which may account for the positive correlations between the frequency of acute exacerbation and age [15]. However, this may also be due to the severity or heterogeneity of the tested patients’ condition.

A case series in 2011 enrolled 16 adult patients with ABPA but without CF and assessed the efficacy of omalizumab by comparing the data before and after one year of treatment, the study demonstrated that omalizumab treatment reduces the frequency of acute exacerbation and the necessary dosage of hormones [16]. Likewise, another study of 14 adult patients with ABPA complicating asthma showed that omalizumab was effective in lowering the frequency of exacerbation and the demand for corticosteroids, as well as improving asthmatic symptoms and pulmonary function parameters [17]. Although consistent with the results of this study, these results were not relevant. However, the frequency of acute exacerbation and the average monthly usage of glucocorticoids after omalizumab treatment were found to be correlated with age and BMI in this study.

Some clinical studies have demonstrated the efficacy of omalizumab in the treatment of severe asthma. A study reviewing 19 clinical trials found that after 16–60 weeks of treatment, the group treated with omalizumab had a lower rate of exacerbation and risk of hospitalization than the placebo group [18]. Furthermore, its immunological mechanism was revealed by a randomized double-blind multicentre study in which omalizumab treatment was effective in reducing the IgE levels in respiratory tract mucosa and blood, as well as the number of eosinophils, for patients with asthma [19].

A recent article investigating the immunological mechanism and efficacy of omalizumab in the treatment of ABPA found that this agent not only reduces FcεR1 and IgE attachment on the surface of basophilic granulocytes but also reduces their susceptibility and maximal responsiveness to *Aspergillus fumigatus*. Even at high IgE levels (2314 ± 2125 IU/mL (mean ± SD)) in adult patients with ABPA complicating asthma, this treatment addressed the immunological mechanism and reduced the frequency of exacerbation without obvious adverse effects. The IgE level of patients enrolled in our study was 1916.58 ± 2083.15 IU/mL (mean ± SD), which is also beyond the range for which omalizumab is recommended. However, conclusions can still be drawn, and this study may serve as evidence supporting the safety and efficacy of omalizumab in patients with ABPA complicating asthma whose serum IgE exceeds the recommended range for omalizumab use [11].

### Limitations

One of the limitations of this study was the lack of pulmonary function tests; such tests were originally planned, but more than half of the patients were considerably resistant due to intolerance. However, some studies have found that omalizumab failed to improve pulmonary function in patients with ABPA complicating asthma [20, 21]. It has even been suggested that pulmonary function may not be an ideal parameter for ABPA and may be a weak indicator of the efficacy of omalizumab [11]. Other limitations include the placebo effect inherent to the retrospective nature of the study and the lack of a comparison group.

## Conclusion

In conclusion, this study, which has the largest sample size to date, demonstrated that omalizumab treatment in adult patients with ABPA complicating asthma was effective in reducing the frequency of acute exacerbation and the necessary dosages of glucocorticoids and other agents. Its efficacy may be related to age and BMI, and its safety profile was favourable. Randomized, double-blind, controlled prospective experiments with a larger number of samples are needed to further validate our findings.

## Data Availability

The data that support the findings of this study are available from the corresponding author upon reasonable request.

## Appendix

See (Tables 4 and 5).

**Table 4:** The case of missing data

Time	Index	Missing amount	Missing per cent
	Duration of asthma, y	2	7.69%
3 M	Eosinophil count, *10^9^cells/µL	3	11.5%
	Total IgE, IU/mL	6	23.1%
6 M	Eosinophil count, *10^9^cells/µL	5	21.7%
	Total IgE, IU/mL	4	17.4%
12 M	Eosinophil count, *10^9^cells/µL	7	36.8%
	Total IgE, IU/mL	8	42.1%

*M* months

**Table 5:** Comparison of multiple interpolations

	Eosinophil count, *10^9^cells/µL		*P* value	Total IgE, IU/mL		*P* value
	Before	After		Before	After	
3 M	0.21 ± 0.22 (0.01,0.81)	0.23 ± 0.21 (0.01,0.81)	0.90	1489.62 ± 1341.47 (171,5004)	1488.31 ± 1169.47 (171,5004)	0.51
6 M	0.25 ± 0.26 (0.01,0.95)	0.26 ± 0.23 (0.01,0.95)	0.63	1750.82 ± 1996.46 (121,6289.2)	1750.10 ± 1805.87 (121,6289.2)	0.65
12 M	0.34 ± 0.36 (0.08,1.3)	0.39 ± 0.27 (0.08,1.3)	0.45	2301.36 ± 2529.07 (328,7787.7)	2302.34 ± 1885.09 (328,7787.7)	0.27

*M* months

## Abbreviations

ABPA: Allergic bronchopulmonary aspergillosis
CF: Cystic fibrosis
IQR: Interquartile range
LABA: Long-acting *β* agonist
SD: Standard deviation
